# Experience-Dependent Structural Plasticity of Adult-Born Neurons in the Aging Hippocampus

**DOI:** 10.3389/fnins.2019.00739

**Published:** 2019-07-17

**Authors:** Mariela F. Trinchero, Magalí Herrero, M. Cristina Monzón-Salinas, Alejandro F. Schinder

**Affiliations:** Laboratorio de Plasticidad Neuronal, Fundación Instituto Leloir, Buenos Aires, Argentina

**Keywords:** dentate gyrus, adult neurogenesis, synaptogenesis, enriched environment, electrophysiology

## Abstract

Synaptic modification in cortical structures underlies the acquisition of novel information that results in learning and memory formation. In the adult dentate gyrus, circuit remodeling is boosted by the generation of new granule cells (GCs) that contribute to specific aspects of memory encoding. These forms of plasticity decrease in the aging brain, where both the rate of adult neurogenesis and the speed of morphological maturation of newly generated neurons decline. In the young-adult brain, a brief novel experience accelerates the integration of new neurons. The extent to which such degree of plasticity is preserved in the aging hippocampus remains unclear. In this work, we characterized the time course of functional integration of adult-born GCs in middle-aged mice. We performed whole-cell recordings in developing GCs from Ascl1^CreERT2^;CAG^floxStopTom^ mice and found a late onset of functional excitatory synaptogenesis, which occurred at 4 weeks (vs. 2 weeks in young-adult mice). Overall mature excitability and maximal glutamatergic connectivity were achieved at 10 weeks. In contrast, large mossy fiber boutons (MFBs) in CA3 displayed mature morphological features including filopodial extensions at 4 weeks, suggesting that efferent connectivity develops faster than afference. Notably, new GCs from *middle-aged* mice exposed to enriched environment for 7 days showed an advanced degree of maturity at 3 weeks, revealed by the high frequency of excitatory postsynaptic responses, complex dendritic trees, and large size of MFBs with filopodial extensions. These findings demonstrate that adult-born neurons act as sensors that transduce behavioral stimuli into major network remodeling in the aging brain.

## Introduction

Aging is characterized by a general decline in cognitive performance including episodic memory formation, which takes place in the hippocampus (HC) (Burke et al., 2012; Leal and Yassa, 2015). Unlike pathological conditions such as Alzheimer’s disease that may involve massive neuronal loss, normal aging may be accompanied by structural, chemical, and functional changes that lead to synaptic and circuit alterations in the HC (Burke and Barnes, 2006; Fan et al., 2017). The granule cell (GC) layer is the information gateway to the HC and exhibits an age-dependent decrease in activity that might impact directly on memory encoding. Input signals collected by dentate GCs pass onto CA3 pyramidal cells via their mossy fiber boutons (MFBs). They contact GABAergic interneurons in the CA3 stratum-lucidum via filopodial extensions from MFBs, which provide feedforward inhibition to pyramidal cells (Acsády et al., 1998; Lawrence and McBain, 2003; Toni et al., 2008; Restivo et al., 2015). With aging, CA3 develops hyperexcitability due to alterations in synapses formed between GCs and GABAergic interneurons (Small et al., 2004; Galván et al., 2011; Marrone et al., 2011; Villanueva-Castillo et al., 2017). These processes are known to affect brain plasticity and function.

In the dentate gyrus, the addition of new GCs throughout life represents an exceptional form of structural plasticity that is thought to play a crucial role in the ability to discriminate similar inputs (a computational model called pattern separation) (Sahay et al., 2011; Kropff et al., 2015). In mice, the production of new neurons drops sharply at 6 months old, mainly due to a decreased proliferation of neural progenitor cells (Kuhn et al., 1996; Kempermann et al., 1998; Morgenstern et al., 2008; Kempermann, 2015; Kuipers et al., 2015). In addition, the fewer GCs that are generated display a marked delay in their morphological maturation (Trinchero et al., 2017), and the extent to which they become morphologically and functionally integrated remains to be elucidated. The decrease in neurogenesis and the delay in GCs integration could both contribute to the behavioral impairments observed in aging mice. Consistent with this notion, aged mice perform poorly in tasks involving behavioral pattern separation, but this deficiency can be improved by chronic physiological stimuli such as voluntary exercise and environmental enrichment, which increase neurogenesis and promote the integration of new GCs both in young and aging mice (Piatti et al., 2011; Wu et al., 2015; Trinchero et al., 2017).

In young-adult mice, a brief experience in an enriched environment (EE) can be sensed by the local hippocampal network and accelerate integration of adult-born GCs, opening the possibility that new GCs encode information about the learned environment at early developmental stages (Chancey et al., 2013; Kropff et al., 2015; Alvarez et al., 2016). In this work, we characterized the time-course of morpho-functional integration and asked if limited exposures to EE would modify integration of new GCs in the middle-aged HC. We show that GCs integrate over 10 weeks, which may be substantially shortened by a limited EE exposure, if it occurs during a restricted neuronal age.

## Materials and Methods

### Mice, Genetic Tagging, and Stereotaxic Surgery for Retroviral Delivery

**Figure 1:**
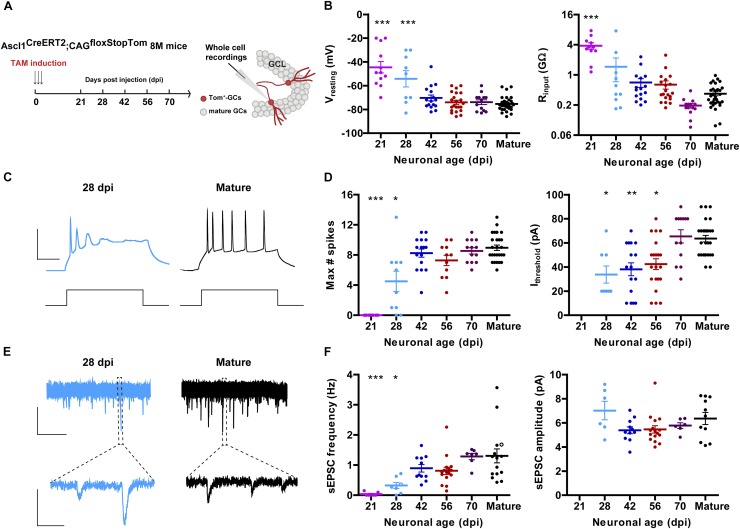
Development of intrinsic excitability and glutamatergic input connectivity in developing GCs from 8 M mice. **(A)** Experimental design. Ascl1^CreERT2^;CAG^floxStopTom^ mice received TAM to label new GCs. Whole-cell recordings were carried out in Tom-GCs at different times after TAM injection in acute slices. **(B)** Resting potential and input resistance from Tom-GCs and unlabeled mature GCs. (^∗∗∗^) denotes *p* < 0.001 compared to mature GCs after ANOVA followed by Tukey’s *post hoc* test (resting potential) and Kruskal–Wallis followed by Dunn’s *post hoc* test (input resistance). Sample sizes (presented as neurons/mice): 12/7 (21 dpi), 9/3 (28 dpi), 16/5 (42 dpi), 22/5 (56 dpi), 13/3 (70 dpi), and 30/17 (mature). **(C)** Representative current-clamp recordings in 28 dpi- (cyan) and mature (black) GCs. Spiking was elicited by depolarizing current steps of increasing amplitude (500 ms, 0–200 pA, 10 pA steps). Scale bars: 50 mV, 50 ms. **(D)** Maximum number of spikes and current threshold for spiking elicited by depolarizing current steps. (^∗^), (^∗∗^), and (^∗∗∗^) denote *p* < 0.05, *p* < 0.01, and *p* < 0.001 compared to mature GCs after Kruskal–Wallis test followed by Dunn’s *post hoc* test. Sample sizes: 15/7 (21 dpi), 10/3 (28 dpi), 16/5 (42 dpi), 11/5 (56 dpi), 13/3 (70 dpi), and 30/17 (mature). **(E)** Representative traces of EPSCs recorded at –70 mV from 28 dpi- (cyan) and mature (black) GCs. Top, time-compressed traces allow the visualization of sEPSC frequency. Bottom, expanded presentations show individual events. Calibration: top, 10 pA, 5 s; bottom, 5 pA, 50 ms. **(F)** Frequency of sEPSC events measured during 120 s (left) and sEPSC amplitude presented as mean value for each cell (right). Open circles correspond to sample traces. (^∗^) and (^∗∗∗^) denote *p* < 0.05 and *p* < 0.001 compared to mature GCs after Kruskal–Wallis test followed by Dunn’s *post hoc* test. Sample sizes: 7/7 (21 dpi), 7/3 (28 dpi), 11/5 (42 dpi), 16/5 (56 dpi), 7/3 (70 dpi), and 15/12 (mature). Horizontal bars denote mean ± SEM.

C57BL/6J *male* mice and genetically modified *male and female* mice were housed at four to five animals per cage in standard conditions. Eight-month-old (8 M) mice were selected because, beyond this age, there is a strong decline in hippocampal neurogenesis that precludes the study of labeled neurons (Morgenstern et al., 2008; Trinchero et al., 2017). Ascl1^CreERT2^ (Ascl1^tm1(Cre/ERT2)Jejo^/J) mice (Kim et al., 2007) were crossed to a CAG^floxStoptdTomato^ (Ai14) [B6;129S6-Gt(ROSA)26Sor^*tm1*4*(CAG-tdTomato)Hze*^/J] conditional reporter line (Madisen et al., 2010) to generate Ascl1^CreERT2^; CAG^floxStopTomato^ mice, which can be used to reliably target adult-born GCs (Yang et al., 2015). Tamoxifen (TAM) administration (120 mg/kg, once a day for 3 days) was carried out in 8 M mice to achieve indelible expression of tdTomato (Tom) in the progeny of Ascl1^+^ progenitor cells. Two to three days before TAM induction or retroviral injection, running mice were exposed to running wheels (one for every two mice) to maximize the number of labeled adult-born GCs. This genetic strategy for labeling adult-born GCs was used in electrophysiological experiments shown in Figure 1, 4.

**Figure 2:**
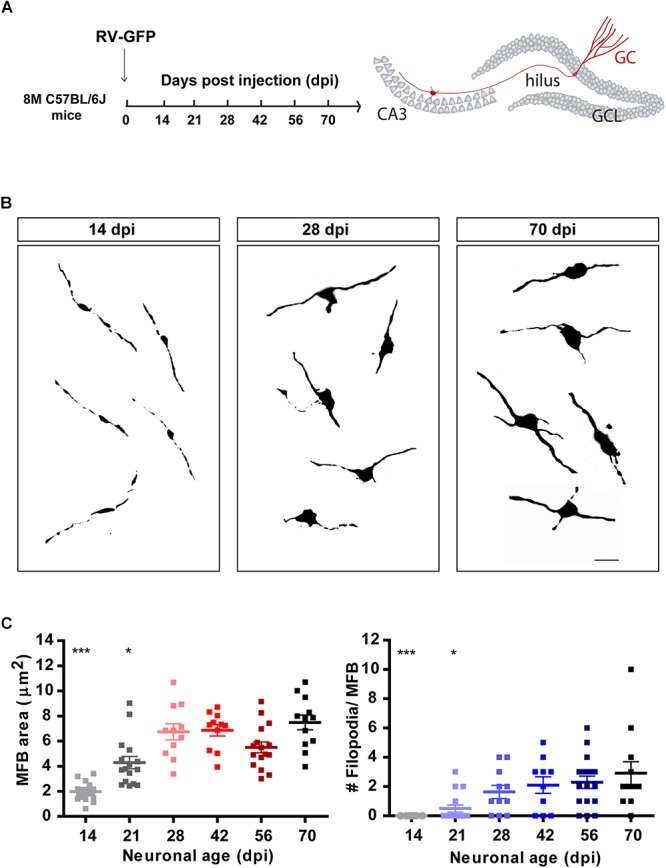
Development of mossy fiber boutons (MFBs) in new GCs. **(A)** Experimental design. RV-GFP was infused in the right dentate gyrus of 8 M mice. Morphology of MFBs in CA3 was analyzed at different times after confocal imaging. **(B)** Representative images of MFBs in CA3 at the indicated times. Scale bar, 5 μm. **(C)** MFB area and number of filopodia. (^∗^) and (^∗∗∗^) denote *p* < 0.05 and *p* < 0.001 compared to 70 dpi GCs after Kruskal–Wallis test followed by Dunn’s test. Sample sizes (neurons/mice): 14/3 (14 dpi), 16/3 (21 dpi), 11/2 (28 dpi), 11/2 (42 dpi), 16/3 (56 dpi), and 12/2 (70 dpi). Horizontal bars denote mean ± SEM.

**Figure 3:**
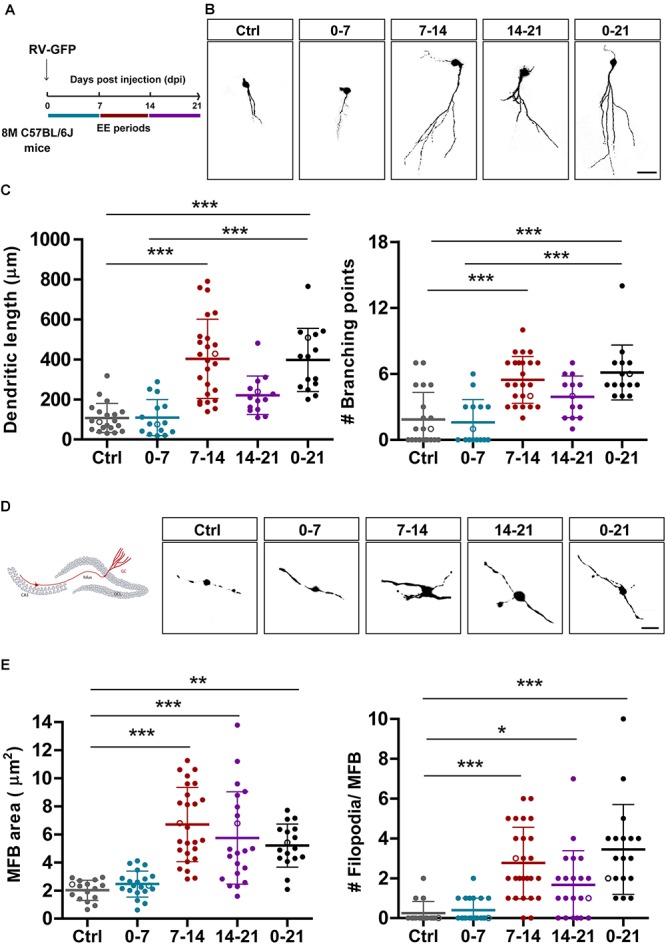
EE triggers structural remodeling of new GCs. **(A)** Experimental design. RV-GFP injection was followed by exposure to 7 days of EE at the indicated 1-week windows or during the entire experiment (0–21). Morphological parameters were analyzed in GFP-GCs at 21 dpi after confocal imaging. **(B)** Representative confocal images of 21-dpi GFP-GCs for the different groups. Scale bar, 25 μm. **(C)** Dendritic complexity (length and branching points) for the different windows of EE exposure. (^∗∗∗^) denotes *p* < 0.001 after Kruskal–Wallis test followed by Dunn’s *post hoc* test. Sample sizes (neurons/mice): 20/4 (Ctrl), 15/3 (0–7), 25/5 (7–14), 14/4 (14–21), and 15/4 (0–21). **(D)** MFB morphology in CA3 was analyzed for all groups, and representative images are shown on the right panels. Scale bar, 5 μm. **(E)** (^∗^), (^∗∗^), and (^∗∗∗^) denote *p* < 0.05, *p* < 0.01, and *p* < 0.001 after Kruskal–Wallis test followed by Dunn’s *post hoc* test. Sample sizes: 16/4 (Ctrl), 20/4 (0–7), 26/5 (7–14), 21/4 (14–21), and 17/3 (0–21). Horizontal bars denote mean ± SEM. Open circles correspond to sample neurons/boutons.

Mice were anesthetized (150 μg ketamine/15 μg xylazine in 10 μl saline per gram), and retrovirus was infused into the septal region of the right dentate gyrus (1.5 μl at 0.15 μl/min) using sterile calibrated microcapillary pipettes through stereotaxic surgery coordinates from bregma (in mm): -2 anteroposterior, -1.5 lateral, and -1.9 ventral. Brain sections were obtained at the indicated times for confocal imaging (Trinchero et al., 2017). Only neurons in the septal dentate gyrus were included in the analysis, corresponding to sections localized from -0.96 to -2.30 mm from the bregma, according to the mouse brain atlas (Paxinos and Franklin, 2001). This retroviral labeling technique using wild-type mice was utilized for structural analysis shown in Figure 2, 3.

Experimental protocols were approved by the Institutional Animal Care and Use Committee of Fundación Instituto Leloir, according to the Principles for Biomedical Research involving animals of the Council for International Organizations for Medical Sciences and provisions stated in the Guide for the Care and Use of Laboratory Animals.

### EE Exposure

At different times after stereotaxic injection (Figure 3A), mice were exposed to an EE consisting of a larger cage (75 cm × 40 cm × 15 cm) with several tunnels and toys (but no running wheel) for 7 or 21 days. In the latter group, EE was modified once a week to maintain novelty. Animals were then perfused for immunofluorescence analysis. Control mice were left in regular cages.

### Immunofluorescence

Immunostaining was done on 60-μm free-floating coronal sections. Antibodies were applied in Tris-buffered saline (TBS) with 3% donkey serum and 0.25% Triton X-100. Immunofluorescence was performed using anti-green fluorescent protein (GFP) (rabbit polyclonal; 1:500; Invitrogen) and donkey anti-rabbit Cy3 antibodies (1:250; Jackson Immuno Research Laboratories).

### Confocal Microscopy

For dendritic length measurements, images were acquired (40×; NA 1.3; oil-immersion) from 60-μm thick sections taking Z stacks including 35–50 optical slices, airy unit = 1 at 0.8-μm intervals (Trinchero et al., 2017). Dendritic length was then measured using the LSM Image Browser from projections of three-dimensional reconstructions onto a single plane in GCs expressing GFP in their soma. Images of GFP-labeled MFBs in the CA3 region were acquired at 0.4-μm intervals (63×; NA 1.4; oil-immersion) and a digital zoom of 6. Area and number of filopodia was analyzed from projections of three-dimensional reconstructions onto a single plane. MFBs that fit the following criteria were selected for quantification: (i) the diameter of the bouton was more than threefold larger than the diameter of the fiber, (ii) the bouton was connected to the mossy fiber on at least one end (Toni et al., 2008). Filopodia were identified as protrusions arising from LMTs (1 μm < length < 20 μm; Acsády et al., 1998). Filopodial extensions were measured by counting the number of protrusions per LMT. For image capture and analysis of morphological properties, all experimental groups under study were blind to the operator.

### Electrophysiology

#### Slice Preparation

Ascl1^CreERT2^;CAG^floxStop-tdTomato^ 8 M mice were anesthetized and decapitated at different times after TAM induction as indicated, and transverse slices were prepared as described previously (Alvarez et al., 2016; Trinchero et al., 2017). Briefly, brains were removed into a chilled solution containing (in mM): 110 choline- Cl, 2.5 KCl, 2.0 NaH_2_PO_4_, 25 NaHCO_3_, 0.5 CaCl_2_, 7 MgCl_2_, 20 dextrose, 1.3 Na^+^-ascorbate, 3.1 Na^+^-pyruvate, and 4 kynurenic acid (kyn). Coronal slices (400 μm thick) from the septal pole containing both hippocampi were cut with a vibratome and transferred to a chamber containing (in mM): 125 NaCl, 2.5 KCl, 2 NaH_2_PO_4_, 25 NaHCO_3_, 2 CaCl_2_, 1.3 MgCl_2_, 1.3 Na^+^-ascorbate, 3.1 Na^+^-pyruvate, and 10 dextrose (315 mOsm). Slices were bubbled with 95% O_2_/5% CO_2_ and maintained at 30°C for >45 min before experiments started.

#### Recordings

Whole-cell recordings were performed using microelectrodes (4–6 MΩ) filled with (in mM): 150 K-gluconate, 1 NaCl, 4 MgCl_2_, 0.1 (ethylene glycol-bis(β-aminoethyl ether)-N,N,N′,N′-tetraacetic acid) EGTA, 10 (4-(2-hydroxyethyl)-1-piperazineethanesulfonic acid) HEPES, 4 ATP-Tris, 0.3 GTP-Tris, and 10 phosphocreatine. Criteria to include cells in the analysis were visual confirmation of Tom in the pipette tip, attachment of the labeled soma to the pipette when suction was performed, and absolute leak current <100 pA at holding potential (*V*_h_). Spontaneous EPSCs were recorded in voltage clamp at -70 mV. Input resistance was assessed by the application of voltage steps of -10 mV in voltage-clamp mode, and spiking was evoked by the injection of current steps (10 pA) in current-clamp configuration after taking the membrane potential to -70 mV. All recordings were performed at room temperature (23 ± 2°C), digitized, and acquired at 10 KHz on a personal computer. Detection and analysis of spontaneous EPSCs were done using a dedicated software package.

### Statistical Analysis

Unless otherwise specified, data are presented as mean ± SEM. Normality was assessed using the Shapiro–Wilk test, D’Agostino–Pearson omnibus test, and Kolmogorov–Smirnov test, with a *p*-value of 0.05. When data met normality tests (Gaussian distribution and equal variance), unpaired *t*-test with Welch’s correction or ANOVA with Bonferroni’s *post hoc* test was used as indicated. In cases that did not meet normality criteria, nonparametric tests were used as follows: Mann–Whitney *U*-test for independent comparisons and Kruskal–Wallis test for multiple comparisons.

## Results

### Electrophysiological Properties of Developing GCs in the Dentate Gyrus of Middle-Age Mice

Aging mice display low levels of hippocampal neurogenesis. We therefore used genetically modified mice Ascl1^CreERT2^;CAG^floxStopTom^ to express Tom in the progeny of neural stem cells and neuroblasts. This allowed us to label a substantial amount of new neurons even in the middle-aged HC to perform electrophysiological recordings (Yang et al., 2015). Mice at 8 M received TAM and acute slices were prepared at different days post-induction (dpi) to assess intrinsic properties and connectivity using whole-cell recordings (Figure 1A). Similarly to what occurs in developing GCs of the young-adult HC, the resting membrane potential was initially depolarized and acquired more hyperpolarized values as neurons matured, by 8 weeks. Input resistance was in the GΩ range for >4 weeks, and reached plateau values at 8–10 weeks (Figure 1B). This high membrane resistance was maintained longer than what was typically reported in young adult mice (Mongiat et al., 2009; Yang et al., 2015), revealing that new CGs may remain highly excitable for longer periods in the aging brain.

Changes in intrinsic membrane properties that accompany neuronal development include the capacity to fire action potentials in a repetitive manner. This change is dictated by the increment in the expression of voltage-gated sodium and potassium channels (Espósito et al., 2005; Mongiat et al., 2009; Gonzalez et al., 2018). New CGs in 8 M mice did not spike until 4 weeks of age, and repetitive spiking reached a plateau level by 6 weeks. Interestingly, the age-dependent increase in the threshold current required to elicit an action potential basically mirrored the decrease in input resistance, and reached a maximum at 10 weeks (Figure 1C,D). Spontaneous excitatory postsynaptic currents (sEPSCs) were recorded to monitor the development of glutamatergic input connectivity in developing neurons. Recordings obtained from 21 dpi neurons displayed no spontaneous synaptic events at all, indicating that functional glutamatergic synapses were still absent in those cells. In fact, sEPSCs begun to be detected from 28 dpi onward and their frequency reached a maximal level at 42 dpi, suggesting that glutamatergic synaptogenesis was completed within this 2-week period (Figure 1E,F). While there was a clear age-dependent increase in sEPSC frequency, the amplitude of the individual events remained unchanged, indicating that the development of glutamatergic connectivity involves a time-dependent increase in the number of contacts but not in the strength of the individual contacts.

### Morphological Development of Mossy Fiber Terminals

Granule cells make glutamatergic excitatory connections onto CA3 pyramidal cells through large MFBs, and recruit GABAergic feedforward inhibition on pyramidal cells via filopodial extensions that extend from MFBs (Acsády et al., 1998; Toni et al., 2008; Sun et al., 2013; Restivo et al., 2015). Confocal imaging was used to measure the size and filopodial number of presynaptic terminals in retrovirally labeled GCs, as indicators of output connectivity. New GCs were identified using retroviral expression of GFP (RV-GFP), which labels a reduced number of cells and thus allows morphological analysis of individual axons and terminals. At 14 dpi, MFBs displayed oval form, a small area of about 2 μm^2^, and lacked filopodial extensions (Figure 2A–C). By 21 dpi, MFB shape became more irregular and displayed filopodial extensions, suggesting that mossy fibers could contact pyramidal cells and GABAergic interneurons in CA3. At 28 dpi, MFBs reached full size and extended two to three filopodia, resembling a mature phenotype.

### Experience Accelerates Input/Output Integration of New GCs in the Aging Hippocampus

In a previous work, we showed that chronic exposure to voluntary exercise or EE accelerate dendritic growth and synapse formation onto adult-born GCs in the *middle-aged* HC (Trinchero et al., 2017). We now investigated if new neurons are sensitive to behavioral experience by EE during restricted developmental windows. We also assessed whether output connectivity is modified by such physiological stimuli. Mice received intrahippocampal injections of RV-GFP and were exposed to EE for 1 week at different intervals or for 3 weeks (Figure 3A). Initially, we analyzed the effect of EE on dendritic length to compare the different short intervals with chronic exposure. Three-week-old neurons from mice exposed to chronic EE showed fourfold increase in dendritic length and in the number of branching points compared to mice in their home cage. Remarkably, exposure of 1 week to EE caused a similar effect, but only when the experience was restricted to the second week of neuronal development (Figure 3B,C). EE also caused a robust increase in the size of MFBs from 2 to 7 μm^2^, but now the effect of the shorter exposure was somewhat larger than the long-term (Figure 3D,E). The formation of filopodial extensions that were absent in GCs from control mice was now boosted by exposure to EE. Interestingly, the period of sensitivity was now 2-weeks long, which suggests that input and output connectivity may follow different activity-dependent mechanisms. Together, these results demonstrate that experience exerts powerful effects on the development and integration of new GCs in the aging brain which, in turn, would favor information flow from the GC layer toward downstream circuits that include GABAergic interneurons and principal glutamatergic cells in CA3.

**Figure 4:**
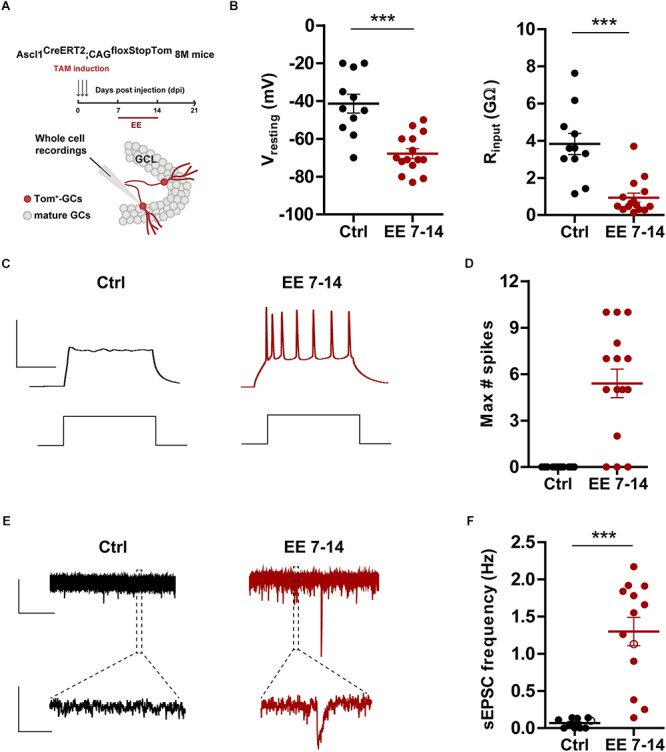
EE triggers maturation of glutamatergic inputs. **(A)** Experimental design. Ascl1^CreERT2^;CAG^floxStopTom^ mice received TAM to label new GCs and one group was exposed EE from 7 to 14 dpi (red bar). Whole-cell recordings were carried out at 21 dpi in Tom-GCs in acute slices. **(B)** Resting potential and input resistance from Tom-GCs exposed to EE or home cage conditions (Ctrl). (^∗∗∗^) denotes *p* < 0.001 after Mann–Whitney test. **(C)** Representative current-clamp recordings. Spiking was elicited by depolarizing current steps of increasing amplitude (500 ms, 0–200 pA, 10 pA steps). Scale bars: 50 mV, 50 ms. **(D)** Maximum number of spikes elicited by step depolarization. Sample sizes (neurons/mice): 11/6 (Ctrl) and 15/5 (EE 7–14). **(E)** Representative voltage-clamp traces depicting EPSCs, recorded at –70 mV from Ctrl (black) and EE 7–14 (red) Tom^+^ GCs. Expanded scale in the bottom shows individual events. Scale bars: 10 pA, 5 s (top), 50 ms (bottom). **(F)** Frequency of sEPSCs measured during 120 s. (^∗∗∗^) denotes *p* < 0.001 after Mann–Whitney test. Sample sizes (neurons/mice): 10/6 (Ctrl) and 13/5 (EE 7–14). Horizontal bars denote mean ± SEM. Open circles correspond to sample neurons/boutons.

The effects observed above suggest that 7 days of EE are sufficient to promote rapid electrophysiological maturation and functional integration of new neurons. To test this possibility, electrophysiological recordings were carried out in 3-week-old GCs using Ascl1^CreERT2^;CAG^floxStopTom^ mice. 8 M mice were kept in control cages or exposed to EE during the second week of development (Figure 4A), the window of EE exposure that resulted in maximal changes in neuronal structure (Figure 3C,E). At the level of intrinsic properties, EE produced a marked hyperpolarization of the resting potential typical of mature GCs and a reduction of input resistance to levels that remained significantly higher than mature cells born during development (EE_7-14_ *R*_input_ = 0.99 ± 0.26 GΩ, *n* = 14 vs. mature GC *R*_input_ = 0.31 ± 0.04 GΩ, *n* = 18, *p* < 0.01 after Mann–Whitney *U*-test) (Figure 4B). In addition, while neurons from control mice did not fire action potentials, GCs from EE mice showed robust spiking, but with immature features (EE_7-14_ *I*_threshold_ = 30 ± 3 pA, *n* = 18 vs. mature GC *I*_threshold_ = 70 ± 3 pA, *n* = 23, *p* < 0.001; and EE_7-14_ Max#spikes = 5 ± 1, *n* = 15 vs. mature GC Max#spikes = 10 ± 1, *n* = 17, *p* < 0.001 after Mann–Whitney *U*-test) (Figure 4C,D). Finally, synaptic integration was quantified as sEPSC frequency, which jumped from no activity in control mice to about 1 Hz in mice exposed to EE (Figure 4E,F), a range similar to that found in mature GCs (Figure 1F). These results demonstrate that short periods of EE boost development and functional integration of new neurons in the *middle-aged* brain, which might in turn participate in learning and memory processes.

## Discussion

It is well known that aging is accompanied by a general deterioration in cognitive functions, but the results presented here reveal that immature GCs endow a remarkable potential for plasticity triggered by a relatively mild experience such as EE. We found that newly generated GCs in middle-aged mice develop slowly but, after sufficient time, reach overall levels of morphological and functional connectivity comparable to neurons born in the developing HC. Previous work has suggested that this delay in neuronal maturation might be due, at least in part, to the overall decrease in the activity of dentate neural circuits that occurs with age, and the concomitant reduction in neurotrophic factors required to promote neuronal growth and connectivity (Trinchero et al., 2017). In fact, a septotemporal gradient of activity in the GC layer has also been linked to a graded maturation velocity in the young-adult dentate gyrus, where new GCs in the more active septal areas develop faster than those in the more silent temporal region (Piatti et al., 2011). Finally, other factors such as oxidative stress and the development of neurons in an inflammatory niche might also contribute to the slow maturation of neurons born in middle-aged mice (Lee et al., 2000; Verbitsky et al., 2004; Hattiangady et al., 2005; Bishop et al., 2010; Guarente, 2014).

During their early development, at about 3 weeks of age, new GCs lacked functional excitatory inputs and structural correlates of synaptic terminals in the target CA3 region. Moving mice from a regular cage to EE for 1 week triggered a switch that allowed to assemble input and output connections, reflected in several-fold increase in dendritic length, larger and more complex MFBs, and the appearance of functional glutamatergic inputs. The integration of new neurons making thousands of afferent and efferent connections imposes a tremendous degree of plasticity to the host circuit (Sailor et al., 2017).

Electrophysiological recordings revealed a delay in the maturation of both passive and active membrane properties as well as input glutamatergic synaptogenesis compared to what has been repeatedly observed in young-adult mice (Overstreet-Wadiche and Westbrook, 2006; Toni and Schinder, 2015; Song et al., 2016). Input resistance remains in the GΩ range for several weeks, which might result in a prolonged window of high neuronal excitability favoring activity-dependent plasticity (Schmidt-Hieber et al., 2004; Ge et al., 2007; Mongiat et al., 2009; Marin-Burgin et al., 2012; Yang et al., 2015). It has been proposed that survival of adult-born GCs relies on the competition for excitatory input connections that maintain a certain level of activity and consequent calcium influx (Tashiro et al., 2006, 2007). It would be relevant to investigate whether the prolonged period of GC hyperexcitability found in the aging brain also results in the extension of the window for activity-dependent survival.

Mossy fiber bouton morphology reached a plateau at 4–6 weeks, achieving 8 μm^2^ and three filopodia per MFB, similar to mature GCs (Toni et al., 2008; Danzer et al., 2009; Wilke et al., 2013). According to their known morphological specialization, MFBs innervate single pyramidal cells on thorny excrescences, and filopodial extensions contact one interneuron on a single contact (Acsády et al., 1998). At the functional level, these structures are expected to convey direct excitation of pyramidal cells and disynaptic feedforward inhibition in the CA3 pyramidal layer (Lawrence and McBain, 2003; Bischofberger et al., 2006). In young adult mice, it was shown that MFBs extend several filopodia at 4 weeks that are then reduced at later neuronal ages, which correlates with a transient enhancement of inhibition in CA3 pyramidal layer elicited by optogenetic stimulation of new GCs (Restivo et al., 2015). While we also observed a transient increase in MFB filopodia at 4 weeks in young-adult mice (not shown), *middle-aged* mice showed a continuous increase in filopodial number that suggests different dynamics in the maturation of new GCs, and predict a graded increase in the recruitment of feedforward inhibition. The functional consequences of this difference between the adult and aging brain will require further electrophysiological investigation regarding the impact of new neurons on the activity of CA3 targets.

Enriched environment enhances HC-mediated learning and plasticity (van Praag et al., 2000; Moreno-Jiménez et al., 2005; Baroncelli et al., 2010; Bergami et al., 2015; Alvarez et al., 2016; Moreno-Jimenez et al., 2019). A brief exposure of young-adult mice to EE was shown to promote new GC integration at an early developmental stage (second week), an effect mediated by the depolarizing action of GABAergic interneurons (Chancey et al., 2013; Alvarez et al., 2016). Here, we showed that a brief exposure to EE in *middle-aged* mice promotes GC integration at the level of input and output connections. With regard to inputs, the effect was observed in dendritic length and in the frequency of sEPSCs only when neurons were exposed during their second week of development. The coincidence in the timing of sensitivity to EE suggests that early signaling mechanisms are preserved in the adult and aging dentate gyrus.

Experience was also shown to promote remodeling and growth of MFBs, even those that belong to fully mature GCs born in the developing brain (Ramírez-Amaya et al., 2001; Galimberti et al., 2006; Gogolla et al., 2007). EE exposure increases MFB size and filopodial number, corresponding to more contacts onto pyramidal cells and interneurons. We now found an exceptional increase in the size and complexity of MFBs with a short stimulus in *middle-aged* animals (Figure 3), where 7 days of EE resulted in MFBs with several filopodia and threefold increase in area. Sensitivity to EE was not limited to the second developmental week but remained for longer, which suggests different mechanisms controlling EE-mediated connectivity in input vs. output synapses. In addition, we also reveal a much higher potential for activity-dependent modification of MFBs belonging to developing GCs than to fully mature neurons.

Our findings highlight a striking contrast in network dynamics in *middle-aged* mice under sedentary conditions compared to mice exposed to EE for a limited period. The aging brain displays a high sensitivity to novel spatial exploration and adult-born GCs are efficient in transducing such behavioral inputs into concrete circuit modifications. Perhaps due to the slow development of new GCs, the effect is stronger than other forms of plasticity reported before in adult mice. Exploiting this endogenous plasticity and understanding its mechanisms will be useful to delay or prevent the continuous cognitive decline occurring in the aging brain.

## Data Availability

The datasets generated for this study are available on request to the corresponding author.

## Ethics Statement

The animal study was reviewed and approved by CICUAL Leloir Instiute.

## Author Contributions

MT, MH, and AS performed the experiments and analyzed the data. MT and AS designed the experiments, wrote the manuscript. MM-S prepared the retroviruses. AS provided the financial support.

## Conflict of Interest Statement

The authors declare that the research was conducted in the absence of any commercial or financial relationships that could be construed as a potential conflict of interest.
